# Optimal Noise Filtering in the Chemotactic Response of Escherichia coli

**DOI:** 10.1371/journal.pcbi.0020154

**Published:** 2006-11-17

**Authors:** Burton W Andrews, Tau-Mu Yi, Pablo A Iglesias

**Affiliations:** 1 Department of Electrical and Computer Engineering, Johns Hopkins University, Baltimore, Maryland, United States of America; 2 Department of Developmental and Cell Biology, University of California Irvine, Irvine, California, United States of America; Institute for Systems Biology, United States of America

## Abstract

Information-carrying signals in the real world are often obscured by noise. A challenge for any system is to filter the signal from the corrupting noise. This task is particularly acute for the signal transduction network that mediates bacterial chemotaxis, because the signals are subtle, the noise arising from stochastic fluctuations is substantial, and the system is effectively acting as a differentiator which amplifies noise. Here, we investigated the filtering properties of this biological system. Through simulation, we first show that the cutoff frequency has a dramatic effect on the chemotactic efficiency of the cell. Then, using a mathematical model to describe the signal, noise, and system, we formulated and solved an optimal filtering problem to determine the cutoff frequency that bests separates the low-frequency signal from the high-frequency noise. There was good agreement between the theory, simulations, and published experimental data. Finally, we propose that an elegant implementation of the optimal filter in combination with a differentiator can be achieved via an integral control system. This paper furnishes a simple quantitative framework for interpreting many of the key notions about bacterial chemotaxis, and, more generally, it highlights the constraints on biological systems imposed by noise.

## Synopsis

Bacterial motility involves successive periods of relatively straight runs, interspersed by tumbles—periods in which the bacteria are reoriented randomly. To move in the direction of chemical gradients—a process known as chemotaxis—cells modulate the duration of the runs. To ascertain whether the direction of the current run is desirable, cells continuously monitor temporal changes in the chemoattractant concentration. However, the decisions can only be based on imperfect information about the environment because binding noise implies that receptor occupancy is a limited measure of the chemoattractant concentration. Bacteria cope by filtering the sensed signal to reduce the effect of this binding noise. Through simulations, Andrews, Yi, and Iglesias demonstrate that there is a particular filter cutoff frequency that achieves optimal chemotaxis. Moreover, using a model of the sensing mechanism, the authors also compute the theoretically optimal system for estimating the chemoattractant concentration from the noisy receptor-occupancy signal. Andrews and colleagues show that these two filtering systems are closely matched, and that their frequency-dependent behavior corresponds to published experimental data. Their results highlight the constraints that noise places on cellular performance as well as demonstrating how cells have evolved to deal with this uncertainty in an optimal fashion.

## Introduction

One of the thrusts of the new area of systems biology is the understanding that biological systems can be studied with many of the tools and theory that are used to analyze man-made systems [1,2]. Biological and engineered systems share many of the same features. One of these is the need to make decisions based on imperfect information about the environment, because the cell relies on noisy sensing [3–7]. The chemotaxis signaling pathway of Escherichia coli serves as an ideal vehicle for studying how biology copes with this form of uncertainty.

Bacteria traverse up gradients of chemical attractants in an efficient manner. This chemotactic behavior is universal in living organisms. Two basic chemotaxis strategies are spatial and temporal sensing [8]. In spatial sensing, cells compare spatial differences in chemical concentration (e.g., front versus back) to determine the direction of the gradient. In temporal sensing, cells detect temporal changes in chemical concentration as they move, thereby providing information about whether the course is favorable.

**Figure 1:**
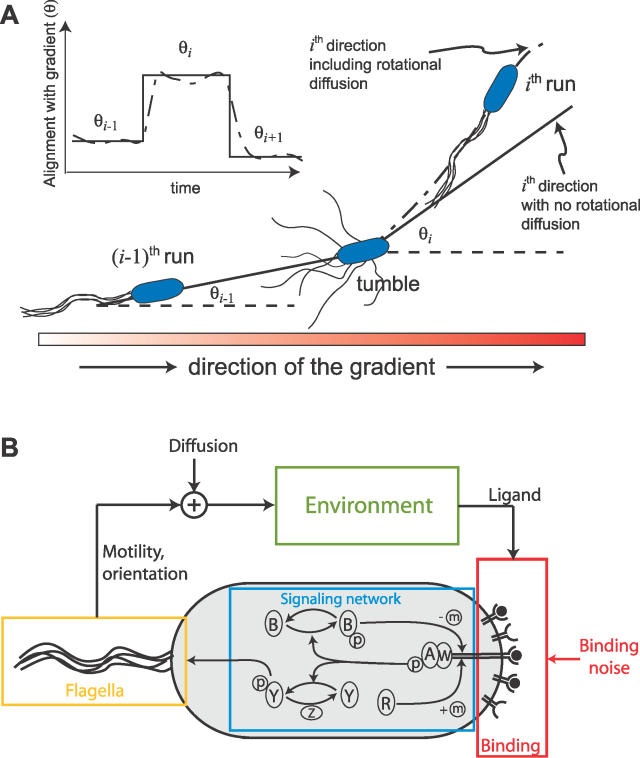
Bacterial Chemotaxis (A) E. coli swim towards nutrients via alternating running and tumbling movements. During each running period, the cell is aligned with the chemoattractant gradient with angle θ*_i_*. These angles change abruptly after each tumble. However, rotational diffusion hinders the ability to swim in straight paths, so that the alignment angle varies stochastically. (B) Chemotactic system. Chemoreceptor complexes contain the proteins CheA and CheW. Ligand–receptor interactions, stochastic in nature, affect the autophosphorylation of the kinase CheA (A), which is capable of transferring its phosphoryl group (P) to the protein CheY (Y). The phosphorylated form of CheY induces clockwise rotation of the flagella, causing tumbling. CheA also transfers phosphoryl groups to CheB (B), an enzyme responsible for demethylation of the receptor complex. Adaptation is achieved via the methylation of the receptor complex by CheR (R). Clockwise rotation of the flagella induces tumbling and a reorientation of the cell while CCW rotation propels the cell forward in a run. Swimming, affected by rotational and translational diffusion, leads the cell to new ligand concentrations in the environment.

The classic work of Berg and colleagues, along with others, have conclusively demonstrated that E. coli employ a temporal sensing mechanism, engaging in a biased random walk consisting of alternating periods of straight runs and random tumbles (Figure 1A, [9]). When the concentration of chemoattractant (*L*) is increasing in time (*dL*/*dt* > 0), the bacteria tend to have longer runs (i.e., the probability of a tumble decreases), thus allowing the bacteria to move up the gradient. If the chemoattractant concentration is decreasing (*dL*/*dt* < 0), then the bacteria are more likely to reorient their direction by tumbling.

The sensing of the chemoeffectors in E. coli is performed by a two-component signal transduction pathway consisting of transmembrane receptors (MCPs, methyl-accepting proteins), receptor methylases (CheR), and demethylases (CheB) that regulate receptor activity, signal transducing histidine kinases, and downstream effectors (e.g., CheY) (Figure 1B, [10,11]). One hallmark of this signaling system is perfect or near-perfect adaptation to a persistent input stimulus, which is a consequence of the temporal differentiation effected by the signaling pathway [12]. It has been demonstrated that this perfect adaptation is robust to dramatic changes in component levels of the pathway, a robustness that can only be achieved through a special type of feedback control, integral feedback [12,13]. However, the adaptation time is quite sensitive to changes in component levels such as concentrations of CheB and CheR [13,14].

**Figure 2:**
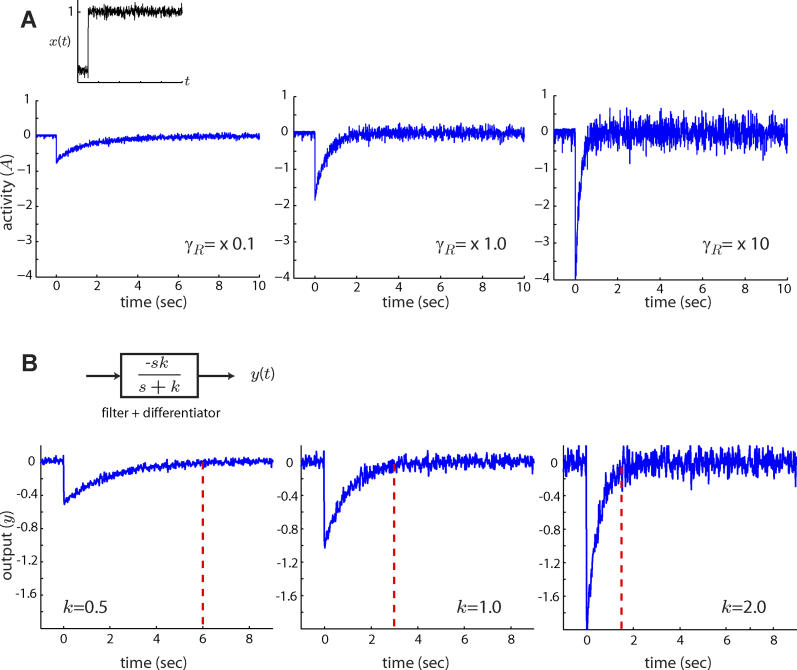
Effect of Adaptation Time on Filtering Capabilities (A) When subjected to an abrupt change in chemoattractant concentration (*x*(*t*)), the output (*A*(*t*)) of the model of the E. coli signaling pathway (Materials and Methods) responds with an initial transient burst which decays exponentially. The adaptation time depends on the component levels of the signaling pathway such as the methylation rate γ*_R_* [13,14]. Faster methylation rates (γ*_R_*) yield shorter adaptation times but result in noisier activity levels. Thus, the filtering capabilities of E. coli are determined by the time it takes to adapt to step inputs of ligand. (B) The adaptation response of E. coli is representative of a system consisting of a low-pass filter (*k*/(*s* + *k*)) coupled with a differentiator (*s*) (inset). A negative gain is used here to mimic the activity response of E. coli to positive changes in ligand [38]. As in the case of the E. coli model, the output of the low-pass filter plus differentiator (*y*(*t*)) is a filtered version of the derivative of the input signal. Smaller filter cutoff frequencies (smaller *k*), which correspond to longer averaging times, yield less noisy outputs. Red dashed lines indicate the time it takes the mean output of the filter to reach 95% of its steady state level. Although longer averaging times help reduce noise, they result in a slower response: the output takes longer to approach zero after the step.

A major challenge of temporal sensing is accurately measuring changes in chemoattractant concentration (*dL*/*dt*) in the presence of noise. Fluctuations, such as those intrinsic to the ligand–receptor binding process, are amplified by this temporal differentiation. A traditional fix in engineering is to use a low-pass filter to remove the high-frequency noise from the lower-frequency signal; the E. coli signaling network implements such a mechanism (Figure 2). The key design criterion is to determine the appropriate cutoff frequency for the filter. In the time domain, this roughly corresponds to selecting the optimal time over which to measure, and average, chemoattractant. The length of time over which the signal is averaged is inversely proportional to the filter-cutoff frequency and determines the adaptation time of a differentiating system to step inputs. This averaging time may also be thought of as a memory length because the cell must “remember” previous values of the input to compute the average. If the averaging or adaptation time is too short, then the noise is not filtered out; alternatively, if the time is too long, then the bacteria cannot detect real changes in the gradient [15,16].

In this paper, we use theoretical tools to study the effects of noise on the ability of a cell to chemotax and, more specifically, on how bacteria cope. We first use simulations to show that adaptation time (filter-cutoff frequency) affects the chemotactic performance of the cell and that there exists an optimal adaptation time that allows cells to chemotax the farthest. Having shown that the adaptation time can make a difference to chemotactic efficiency, we conjecture that this effect is predicated on the ability of the cell to estimate its environment accurately in the presence of noise. To test this, we use theoretical tools to determine which filter-cutoff frequency leads to optimal detection of temporal changes in chemoattractant concentration. The signal to be estimated is ligand concentration, and noise arises from the discrete nature of ligand–receptor interactions during binding. We demonstrate that the theoretically obtained cutoff frequency for optimal estimation has the same dependence on external parameters (e.g., noise and rotational diffusions levels) as the adaptation time that achieves optimal chemotaxis in simulation, implying that optimal ligand estimation is necessary for optimal chemotaxis. Finally, we illustrate how integral feedback control, a known characteristic of the bacterial signaling network [12], implements robust temporal differentiation in which a first-order filter is in series with the differentiator. Thus, we show that the signal transduction pathway found in E. coli is an implementation of the optimal sensing mechanism we derive theoretically. This fact is further verified by quantitative comparison of our results with published models of the signaling network and experimental data.

## Results

### Adaptation Times Affect Chemotaxis

We first examined the effect of adaptation time on chemotaxis through simulation. To this end, we developed a model of the signal transduction network of E. coli (Materials and Methods). This model is based on the receptor-modification model of [17] and other more recent models that include receptor clustering and recreate the experimentally observed chemotactic behavior faithfully [18,19]. A simplified system consisting of a differentiator coupled to a first-order filter captures the primary features of this model: the cell averages the measurement of ligand over a period of time, and then differentiates to determine the quality of the current run direction (Figure 2, Materials and Methods). This approximation of the signaling pathway allows us to adjust the filter's cutoff frequency, mimicking the effect of changes in the concentrations of signaling components on adaptation time, while keeping other signaling characteristics constant (Figure 2B). The system's gain was selected to reflect the gain of a model of the system. The simulations are 2-D and include the run/tumble decision, the motion of the bacterium, as well as the physical implementation of diffusion and noisy signaling. A 1-D linear gradient of attractant was applied, and the chemotactic performance was measured by the average migration distance up the gradient over a period of time. Other key parameters in our simulations were the rotational diffusion constant and the measurement noise variance (Materials and Methods).

**Figure 3:**
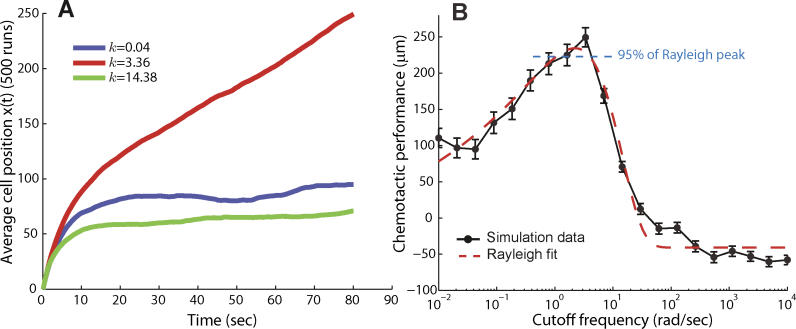
Effect of Adaptation Time on Chemotactic Efficiency Chemotaxis was simulated by assuming that the signaling pathway was approximated by the system of Figure 2B. (A) Different choices of *k,* and hence different adaptation times, result in varying chemotaxis efficiency. For example, cells with cutoff frequencies of *k* = 0.04 rad/s and *k* = 14 rad/s moved approximately 90 μm and 70 μm along the chemoattractant gradient. In contrast, a cell with a cutoff frequency of 3.4 rad/s moved approximately 250 μm along the gradient. (B) These simulations were repeated for a large range of cutoff frequencies. The resulting frequency-dependent chemotactic performance was fitted with a Rayleigh function to estimate the optimal cutoff frequency for chemotaxis (red dashed line). Chemotactic performance was based on the final position along the gradient after 80 s. Each point represents the average of 500 simulation runs, and vertical bars indicate mean plus or minus standard error of the mean. Rotational and translational diffusion coefficients of 0.16 rad^2^/s and 2.2 × 10^−1^ μm^2^/s, respectively, were used. Nominal parameters used: *u* = 20 μm/s, *R_T_* = 2.5 μM, *K_d_* = *k*_−_/*k*_+_ = 100 μM, *L*_0_ = 0.01 μM, and *g* = 0.2 μM/ μm.

Our simulations indicate a biphasic response: cells with a particular filter-cutoff frequency move farther up the chemoattractant gradient than cells with either higher or lower frequency cutoffs (Figure 3A). Moreover, the range of frequencies for which optimal chemotaxis efficiency is observed was narrow (Figure 3B). For example, an approximately 2-fold increase in cutoff frequency from the optimal (from 3.4 to 7.0 rad/s) significantly decreased the distance traveled (249 ± 13 versus 169 ± 10 μm, SEM, *p* < 10^−5^ using Student's t-test). Similarly, a 4-fold decrease in the cutoff frequency (from 3.4 to 0.8 rad/s) also reduced the distance traveled, though less significantly (213 ± 15 μm, SEM, *p* < 0.04). These trends are also observed in signaling models that included higher order rolloff (Figure S1). These data illustrate that to achieve optimal chemotaxis, it is necessary to have optimal or near-optimal noise-filtering capabilities.

**Figure 4:**
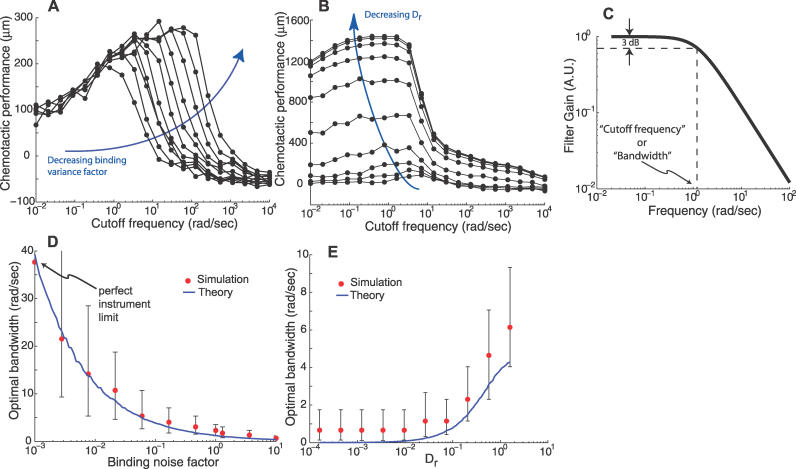
Noise and Rotational Diffusion Effects on Chemotaxis and the Optimal Filter (A) The effect of the measurement noise on the optimal cutoff frequency for chemotactic performance was studied by varying the measurement noise (by multiplying the binding variance by a factor) by several orders of magnitude and determining, as in Figure 3B, the optimal cutoff frequency. All cases showed a biphasic response. Decreasing the binding noise level results in higher optimal chemotactic cutoff frequencies and improved chemotaxis. (B) Similarly, the effect of rotational diffusion on chemotactic performance was studied by varying the diffusion coefficient by several orders of magnitude. Decreasing rotational diffusion increases the chemotactic efficiency, but decreases the optimal cutoff frequency. (C) The optimal filter for chemoattractant estimation was computed and its frequency-dependent magnitude plotted for a specific combination of model parameters. Its shape can be approximated by a first-order filter where the cutoff frequency is the frequency at which the gain is 0.707 (−3dB point) that of the zero frequency gain. (D,E) The noise and rotational diffusion dependence of the optimal cutoff frequencies for chemotaxis obtained in A and B was compared with the optimal cutoff frequencies for chemoattractant estimation predicted by the optimal filter theory. The vertical bars indicate cutoff frequencies that yield chemotactic performances of 95%–100% of the maximum of the Rayleigh curve fitting (see Figure 3B). Simulation and optimal filter parameter values were as in Figure 3.

To determine how external parameters affect the optimal filtering conditions and how they relate to chemotaxis performance, we repeated our simulations for a wide range of binding noise levels and rotational diffusion coefficients (Figure 4A and 4B). In all cases the cells exhibited the same biphasic frequency dependence, indicating that the existence of an optimal filter-cutoff frequency was a robust feature of the network. However, the specific cutoff frequency at which optimal chemotaxis was achieved was parameter-dependent (see below).

### Optimal Filtering in E. coli

The simulations above indicated the existence of specific adaptation times that lead to optimal chemotaxis, and that these adaptation times are dictated by the cutoff frequency of a low-pass filter. To investigate this further, we used tools from estimation theory to deduce the optimal filter that best separates the true ligand concentration from the noise induced by ligand–receptor binding. We assumed that E. coli is swimming in an environment in which the concentration of the chemoattractant at point *z* is *L*(*z*). Moreover, the movement of the bacterium leads to a time-varying change in concentration in its neighborhood: *L*(*z*(*t*)). The bacterium must track how *L*(*z*(*t*))—the “signal” of the system—changes over time to determine whether the current swimming direction is favorable (*dL*/*dt* > 0) or unfavorable (*dL*/*dt* < 0). This determination and its effect on cell movement, achieved via the signal transduction network [20,21], leads the cell to new ligand concentrations *L*(*z*(*t*)), thus completing the feedback loop between the cell and its environment (Materials and Methods).

In practice, the cell can only determine an estimate of *L*(*z*(*t*)) based on the number of receptor–ligand complexes on the cell surface. Moreover, this estimate is corrupted by the presence of noise. Sources of noise include: (i) stochastic binding dynamics of ligand to receptor (ligand binding noise [22]), and (ii) counting fluctuations associated with the diffusion of a small number of ligand molecules into a finite volume (ligand diffusion noise [15,23,24]). We assume that the concentration of ligand-bound receptor complexes is *C*(*t*) + *v*(*t*) where *C*(*t*) is the concentration of receptor complexes in the absence of noise and *v*(*t*) is a noise term due to ligand–receptor binding. We now ask the following question: from a signal processing perspective, what is the optimal filtering mechanism that best estimates *L*(*z*(*t*)) from the noisy observation *C*(*t*) + *v*(*t*)? To answer this, we first made several assumptions about the cell's environment.

We assumed a 2-D environment with linear ligand gradient *g* (μM/μm). Then *dL*/*dt* = ∂*L*/∂*z*·∂*z*/∂*t* = *gu*cos(θ(*t*)), where *z* is cell position, *u* is the magnitude of the cell's velocity, and θ(*t*) is the orientation of the cell at time *t* with respect to the gradient. We treated θ(*t*) as a zero-mean white random process with variance 2*D_r_*τ, where τ is an average length of a bacterial run, and *D_r_* is the rotational diffusion coefficient. This implies that *dL*/*dt* = *w*(*t*), where *w*(*t*) is a white random process with variance *g^2^u^2^*var(cos(θ(*t*))). Thus, changes in the signal *L*(*z*(*t*)) of the system are affected by rotational diffusion: larger *D_r_* yields greater signal fluctuations.

The optimal estimate of *L*(*t*) given the observed signal *C*(*t*) + *v*(*t*) can be found using the Kalman filter, an algorithm developed to detect and separate signals in the presence of random, unwanted noise, that is used widely in engineering navigation and guidance systems [25,26]. We computed the optimal estimator numerically for the state *L*(*z*(*t*)) under the assumption that *C* + *v* is observed (Materials and Methods). The optimal estimator is a low-pass filter: at low frequencies, the system response does not depend on frequency; at higher frequencies, a frequency-dependent dropoff, inversely proportional to frequency, and characteristic of a first-order low-pass filter, is seen (Figure 4C). Moreover, there was good agreement between the theoretical optimal cutoff frequency for ligand estimation and the filter-cutoff frequency required for optimal chemotaxis in the simulations. We repeated this computation for a range of noise levels and rotational diffusion coefficients. There was a striking similarity in the observed trends. For example, the optimal cutoff frequency for both chemotaxis and ligand estimation decreases as the level of binding noise in the system increases (Figure 4D). In contrast, increasing the level of rotational diffusion in the simulations led to higher optimal cutoff frequencies for chemotaxis; a trend that was also consistent with the cutoff frequency of the optimal ligand estimator (Figure 4E). This consistency between the theory and simulations argues that optimal ligand concentration estimation as defined by the Kalman filter is necessary for optimal chemotaxis in our simulations.

**Figure 5:**
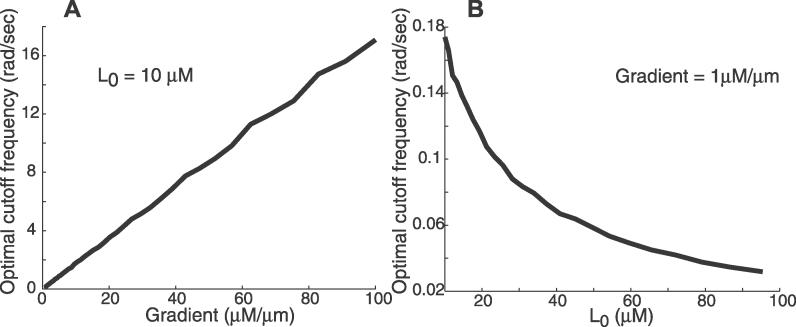
Environmental Factors Influence the Optimal Cutoff Frequency for Chemoattractant Estimation The optimal filter for chemoattractant estimation was computed for a range of gradients (A) and mean concentrations (B). Nominal parameter values were as in Figure 3.

To investigate the dependence of the optimal cutoff frequency for chemoattractant estimation on environmental conditions, we repeated our computations for different levels of mean ligand concentration and varying spatial concentration gradients (Figure 5). In all cases the frequency response of the optimal filter was that characteristic of a first-order low-pass filter. However, the cutoff frequencies varied from one condition to another. In particular, cutoff frequencies increased with increasing chemoattractant gradients, but decreased as the mean level of ligand increased. These variations suggest that chemotactic signaling systems need to be tuned to their environment for optimal estimation of the environment.

### Quantitative Comparisons to Experimental Observations

**Figure 6:**
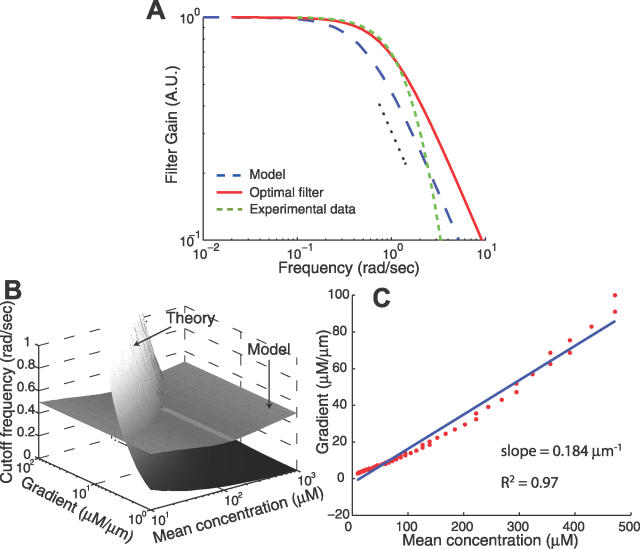
Frequency Dependence of the Signaling Response (A) The frequency-dependent filtering responses for the optimal filter (red solid line) and experimental data (green dashed line; [20]). The experimental data, obtained as the Fourier transform of the response of cells to an impulse of chemoattractant [20] and adjusted to remove the differentiator and downstream phosphorylation cascade (Materials and Methods), also exhibits the characteristics of a low-pass filter. Parameters used for the theoretical filter shown here are *L*_0_ = 1 μM, *g* = 1.5 μM/μm, and *u* = 20 μm/s. Also included is the frequency response of the model (blue dashed line) linearized about a ligand input of *L*_0_ = 1 μM. The dotted black line shows a dependency of (frequency)^−1^. (B) The predicted optimal cutoff frequency is compared with that of the filter for a range of chemoattractant gradients and mean concentrations. The line through which both surfaces intersect represents the chemoattractant profiles for which E. coli filters out disturbances optimally with respect to the parameters used for the model. (C) Plot of the concentration gradient against mean concentration for the points where the surfaces in (B) intersect. The linear dependence suggests that E. coli is conditioned for optimal filtering in chemoattractant concentration profiles of constant relative gradient.

We next determined the degree to which the cutoff frequencies of the optimal filter for chemoattractant estimation matched the frequency-dependent behavior observed experimentally. To this end we used published data describing the behavior of the E. coli chemotactic system [20,27], adjusted to comprise only the primary filtering component by removing the differentiator and the phosphorylation cascade modeled in [28] (Materials and Methods). Additionally, we used our full model of the E. coli signaling pathway as a proxy for the wild-type E. coli chemotaxis behavior. We linearized the model (valid for moderately large step changes in ligand, Materials and Methods) and computed the corresponding low-pass filter component to compare with our theoretical results. Linearizations of other models [17] also revealed similar low-pass filtering characteristics (Materials and Methods). Interestingly, the cutoff frequencies observed from the experimental data and calculated from the model matched reasonably well the theoretical optimal value for a particular gradient and ligand concentration (Figure 6A). This agreement suggests that the chemotaxis signal transduction pathway in E. coli is acting as an optimal filter. It should also be noted that other experimental evidence suggests lower cutoff frequencies (adaptation times on the order of several minutes) [13]. This may be due to strain differences or saturation of the methylation machinery from large ligand stimulants.

We computed the cutoff frequencies of the model for a wide range of chemoattractant gradients and mean concentrations and compared these with the theoretically derived optimal estimator cutoff frequencies (Figure 6B). We found that the two sets did not intersect for all ligand-gradient combinations. However, we did find a range of combinations in which the model filter matches the optimal cutoff frequency (Figure 6C). Remarkably, this combination exhibits an approximately linear dependency (slope 0.184 μm^−1^, R^2^ = 0.97), suggesting that the filtering characteristic of E. coli is optimized for environments where the relative gradient of chemoattractant is constant. This dependency on the relative gradient is reminiscent of earlier results in other organisms, including amoebae [29–31] and yeast (S. Paliwal et al., unpublished data), that focused on chemoattractant gradient sensing.

## Discussion

Previously, Berg and others have argued that the choice of a time over which the bacterium integrates the observed signal to determine the tumbling decision balances the need to average fluctuations in the measurement of ligand levels with the ability to track rapid changes in direction caused by rotational diffusion of the cell [32]. We have constructed a simple mathematical framework to capture these ideas. The notion of integration time, or adaptation time, is captured by the cutoff frequency of the filter that attenuates stochastic disturbances in the sensing mechanism. Using simulations, we demonstrated that chemotactic efficiency is quite sensitive to this filter-cutoff frequency (Figure 3). In particular, we showed that there is a frequency for which optimal chemotaxis can be achieved, and that this frequency is dependent on external parameters (Figure 4). Next, using theoretical tools, we computed the filter that best estimates the ligand concentration in the face of noisy ligand–receptor complexes. We found strong agreement between the cutoff frequencies associated with optimal chemotaxis and optimal chemoattractant estimation (Figure 4D and 4E).

**Figure 7:**
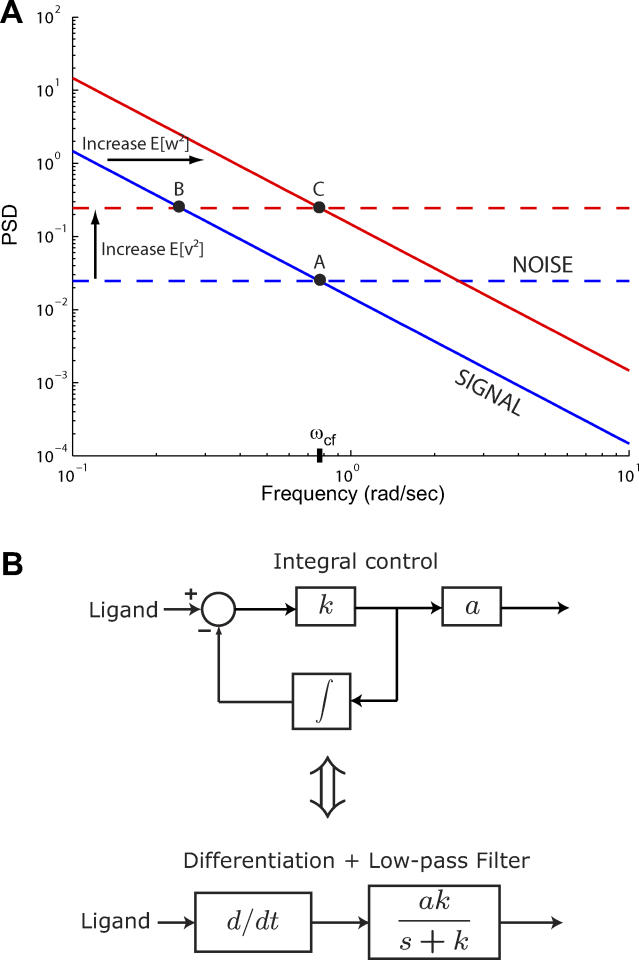
Optimal Filtering Cutoff Frequency Is Determined by the Signal and Noise (A) Insight into the relevant features of the optimal estimator can be obtained by considering a simplified model that assumes the cell measures *L* + *v,* where *L* is the true ligand concentration. The system *dL*/*dt* = *w* acts as an integrator and has signal PSD dependent on the rotational diffusion coefficient (blue solid line). The observed signal *L* + *v* includes the effect of the binding noise *v* which is assumed to be a white noise process (blue dashed line). For this model, the optimal filter for the estimation of *L*, given *L* + *v,* is a first-order, low-pass filter with a cutoff frequency (ω*_cf_*) related to the covariances of *w* and *v*: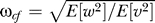 (Protocol S1). Graphically, this is determined by the intersection of the signal and noise PSDs (point A). Increasing the noise variance (red dashed line) decreases the optimal cutoff frequency because the filter must become more restrictive to eliminate the additional noise (point B). If the signal PSD is then increased (red solid line), for example, by increasing the effect of rotational diffusion, the cutoff frequency increases (point C). Parameters used for point A: *u* = 20 μm/s, *D_r_* = 0.16 rad^2^/s, τ = 1 s, *R_T_* = 2.5 μM, *k*_−_/*k*_+_ = 100 μM, *g* = 0.03 μM/μm, and binding is assumed at steady state with a ligand value of *L*_0_ = 1 μM. (B) Block diagram representation of the chemotactic system. A system using an integral control feedback mechanism (top) is functionally equivalent to one consisting of the series connection of a differentiator and a first-order low-pass filter. In the chemotaxis pathway, this subsystem is followed by a conversion function of CheYp to the running bias.

The optimal cutoff frequency for chemoattractant estimation represents the averaging time that best compromises the tradeoff between noise attenuation and signal amplification. This is illustrated in Figure 7A, which highlights the contributions of the noise and signal power spectral densities (PSDs) to this tradeoff. As binding noise increases, the noise PSD shifts upward, and the optimal cutoff frequency (ω*_cf_*) decreases, thus yielding a longer time over which to average ligand measurements so that the additional noise may be attenuated. This trend was seen in the chemotaxis simulations (Figure 4D). As fluctuations of the signal increase (e.g., by increasing the effect of rotational diffusion), the signal PSD shifts upward, and ω*_cf_* increases to yield shorter averaging times that are necessary for observing the more rapidly changing signal; see Figure 4E.

It should be noted that while we have considered binding fluctuations as the measurement noise that results solely from the stochastic dynamics of ligand–receptor interactions, the total noise that contributes to uncertainty in ligand measurement also depends on ligand diffusion [15,24]. Diffusion of ligand molecules in the environment affects their interaction with receptors positioned on the cellular membrane and provides a physical limitation to the accuracy of cell measurements. Estimates of the variance of measurement uncertainty due to ligand diffusion yield measurement fluctuations on the order of 10^−5^ μM^2^ [15,24]. The variance in the amount of bound receptor complexes due to binding dynamics can be quantified at steady state and is on the order of 0.1 μM^2^ [4,33]. Thus, the binding-dynamics noise component overwhelms any uncertainty from ligand diffusion and so we only consider noise from ligand–receptor dynamics in our analysis. However, ligand diffusion provides a limit (the perfect sensor limit) which restricts the degree to which the optimal filter cutoff frequency can increase in response to decreasing binding-dynamics noise (Figure 4D).

We have shown that a low-pass filter is necessary for optimal chemotactic performance of the bacterial system in the presence of noise. Integral control provides an important unifying theme in this work. An integral feedback control system implements a differentiator in combination with a first-order low-pass filter (Figure 7B). This implementation is robust because the central properties of this differentiator depend only on the integral feedback controller (e.g., receptor methylation/demethylation dynamics) and not on the rest of the system (e.g., phosphorylation dynamics of cascade, interaction of CheY-P with motor, etc.). Furthermore, in the context of integral feedback control, the connection between the kinetics of receptor methylation/demethylation, adaptation time, integration time, and the filter breakpoint becomes apparent. Indeed, one can view robust perfect adaptation as a consequence of robust temporal sensing realized via integral control.

The theory and simulations offer several important predictions. First, the activities of the receptor methylase CheR and the demethylase CheB determine the breakpoint of the chemotactic differentiator. For example, increasing the level/activity of CheR causes the breakpoint to shift to larger frequencies and, correspondingly, shorter adaptation times. This trend is observed in the experimental results of [13] and the model of [14]. More importantly, if, as we theorize, E. coli has evolved towards an optimal filter, then moving the breakpoint by altering the methylation/demethylation dynamics should cause impaired chemotaxis performance. Based on our results, we predict that these cells could not chemotax as far up a gradient as cells with wild-type expressions of CheB and CheR. Second, we expect the optimal breakpoint to depend on certain environmental/cellular conditions. In particular, optimal cutoff frequencies may differ by an order of magnitude depending on mean chemoattractant concentrations or the gradient steepness. Also, a larger cell (bacterium) possessing a smaller rotational diffusion coefficient is expected to have a longer optimal integration time (all things being equal). Likewise, slowing the receptor–ligand binding dynamics should increase the measurement noise, leading again to a longer optimal integration time. These parameter-dependency issues suggest the need for further experimental data with a focus on correlating the frequency-dependent responses of both mutant and wild-type cells under varying environmental conditions to their chemotactic efficiency. Another issue for future study is the effect of the filter gain. Because the aim of this work is establishing the existence of an optimal low-pass filter for chemoattractant estimation and chemotaxis, we have focused on frequency dependencies and the optimal cutoff frequency and neglected optimal filter gains.

Finally, our results also highlight the fragile nature of the chemotaxis system by demonstrating how sensitive chemotactic performance can be to changes in adaptation times. In fact, the experiments that definitively showed that the property of adaptation was robust also demonstrated that the adaptation times were not: changes in CheB and CheR expression levels cause significant changes in adaptation time [13,14]. As stated above, our results predict that these cells would not chemotax as efficiently, and point towards a potential fragility of the system [34]. However, recent experimental results demonstrate that within a population of cells there is large variability in behavior and that this is controlled by the methylation process [5]. This variability can be exploited by the population of cells to ensure that at least some of the cells would chemotax efficiently, thus providing a means by which the population of cells can cope with the fragile nature of the individual's signaling systems.

## Materials and Methods

### Model of E. coli signaling network.

**Figure 8:**
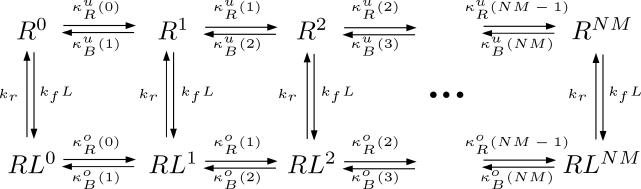
Model of E. coli Signaling Network A model of the bacterial signaling network is shown for a cluster of *N* receptors with *M* methylation sites each. The receptor cluster can exist in a ligand bound or unbound form, each with up to a total of *NM* sites methylated. The methylation (demethylation) rate of a particular state is proportional to the probability of the state being inactive (active).

We developed a model of the E. coli signal transduction network combining the basic receptor-modification model of Barkai and Leibler [17] with more recent models of receptor clustering [19,35]. We assume the cell senses ligand with a cluster of *N* receptors, each with *M* methylation sites. In general, each receptor in the complex can exist in either an “on” or “off” state and can be either ligand-bound or ligand-unbound. The probability of a receptor being on depends on the free energy difference between the on and off states of the receptor and on that of neighboring receptors. Here, we assume the entire cluster is either occupied or unoccupied by ligand and that the free energy difference between the on and off states of each receptor in the cluster is dictated by the cluster's occupancy (not the occupancy of the individual receptors). We treat the cluster as one system with 2(*NM* + 1) states: ligand-unbound with *j*∈{0,1,2,...,*NM*} sites methylated (denoted *R^j^*) or ligand-bound with *j* sites methylated (denoted *RL^j^*); see Figure 8.

The probability of the entire cluster being on when *j* sites are methylated must account for each of the possible microstates that may form this configuration. For example, a cluster of four receptors with a total of three sites methylated can arise when the individual receptor methylation levels are either (3,0,0,0) (i.e., one receptor has three sites methylated and the remaining three receptors have none), (2,1,0,0), or (1,1,1,0). Note that ordering does not matter. We assume the probability of the cluster with *j* methylated sites being on is the average of the probability of each admissible microstate (one with *j* total methylated sites for the cluster) being on:

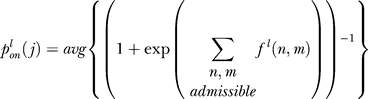
where the average is over all microstates that consist of *j* total methylated sites, *l* indicates ligand occupancy of the cluster (*l* = *u* for ligand-unoccupied or *l* = *o* for ligand-occupied), *f^l^*(*n*,*m*) is the free energy of receptor *n* when *m* of its *M* sites are methylated, and the summation in the denominator is over all receptors and individual receptor methylation levels that form the admissible microstate. In our example, the probability of the cluster with three methylated sites being on is

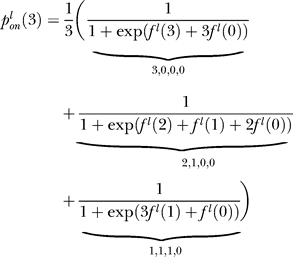
where we have assumed that all of the microstates in the configuration are equally probable. Moreover, we assume that the energy difference of each ligand-unbound/bound process is equal regardless of the receptor, thus allowing us to drop the index *n*.

Only inactive states may be methylated, and only active states may be demethylated. Thus, a state with *j* sites methylated is (further) methylated at a rate

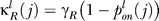
where *γ_R_* is the methylation rate constant. The demethylation rate of a state with *j* sites methylated is

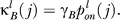



In general (with obvious exceptions for *R* ^0^, *RL* ^0^, *R^NM^*, and *RL^NM^*), the differential equations describing the rate of change of concentration of states *R^j^* and *RL^j^* are

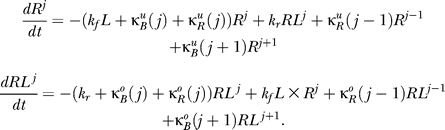



Total activity of the cluster is

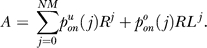



### Model validation.

**Figure 9:**
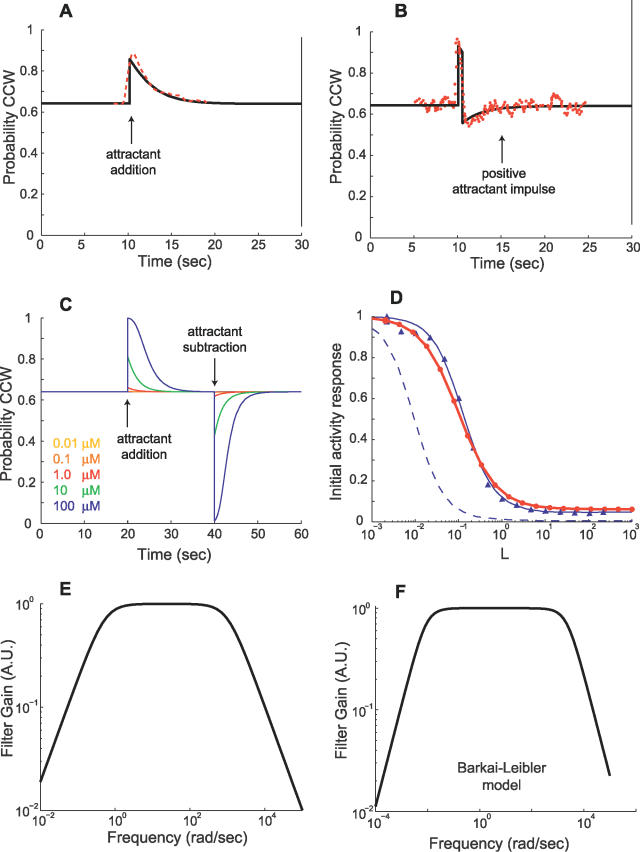
Validation of E. coli Signaling Network Model (A) Response of the E. coli signaling network model is plotted for a 0.1-mM step increase in chemoattractant concentration from 0.2 mM at 10 s. For comparison with experimental data, activity from the model is converted to the probability of CCW flagella rotation by means of a Hill function (Materials and Methods). A transient response after an initial increase in CCW probability adapts to the pre-stimulus level. Adaptation times are similar to experimentally obtained data [20,27] (red dashed line shows data from Figure 2 of [27]). (B) Response to a positive (+0.2 mM) chemoattractant impulse of width 0.5 s is also consistent with experimentally observed behavior [20,27] (red dots show data from Figure 1 of [27]). (C) The initial response to addition (removal) of attractant increases (decreases) with increasing change in attractant concentration. Adaptation times for attractant addition are longer than that for removal as observed [17]. (D) For step inputs, normalized peak activity of the model (1 – Δ*A*/*A_ss_,* where Δ*A* is the change in activity from steady-state level *A_ss_*) exhibits sensitivity to the size of the step (red line with dots is a guide to the eye). This sensitivity response closely matches data from the “small lattice” model of [36] (blue line with triangles is a fitted sigmoid function). The blue dashed line indicates experimental data from [27] as plotted in Figure 4 of [36] (MeAsp input concentration of [27] is adjusted to that of aspartate that yields an equivalent receptor occupancy). As discussed in [36], the nonzero baseline of our model for large step inputs is due to a fraction of receptors in the cluster always having a nonzero probability of being active. (E,F) Frequency responses of the E. coli signaling network model and the model from [17], linearized about a ligand input of *L*_0_ = 1 μM, reveal low-pass characteristics consistent with observations in [27].

We compared a variety of different response characteristics of the model to those observed experimentally (Figure 9). Step increases in chemoattractant induce an initial transient decrease in receptor activity, corresponding to an increase in the probability of counterclockwise (CCW) flagella rotation, that decays exponentially to its pre-stimulus steady-state level (Figure 9A). This behavior is consistent with responses observed experimentally [13,20,27] and in previously published models [14,17,21,35–37]. The response to impulses of chemoattractant exhibit an initial spike in CCW probability followed by a decrease below the pre-stimulus level that then increases exponentially back to the pre-stimulus level (Figure 9B). Step and impulse responses agree closely with experimentally observed responses from [27]. Step-decreases in chemoattractant induce a response similar to step-increases with an initial decrease in CCW probability instead of an increase (Figure 9C). Both the adaptation time (the time required for the step response to return to its pre-stimulus level) and response magnitude increase with increasing step size and are asymmetric with respect to positive and negative steps (Figure 9C, [17]). The peak activity response to step inputs exhibits high sensitivity to the size of the step (Figure 9D), as has been observed experimentally [38,39], and is seen in other published models [18,19,35,36,40]. Sensitivity of our model closely matches that of [36].

The model parameters used are *N* = 4, *M* = 4, *k_f_* = 0.01 μM^−1^ ms^−1^, *k_r_* = 1 ms^−1^, γ*_R_* = 1.2 s^−1^, and γ_B_ = 2 γ*_R_*. Free energy differences used are given in Table 1.

**Table 1:**
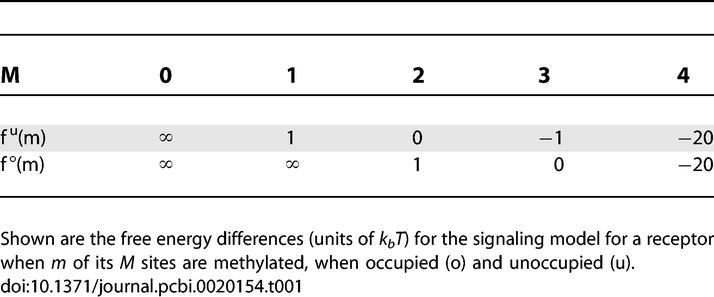
Free Energy Differences

### Model linearization and frequency response.

**Figure 10:**
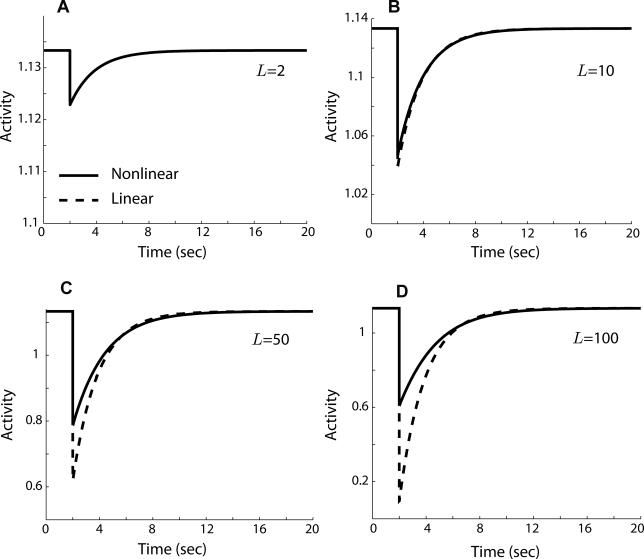
Step Response of the Linearized Signaling Network Comparison of the step response of the E. coli signaling network model to the response of the linearized form of the model shows that the linearization is valid for even moderately large step changes in attractant. Response shown is for a step ligand change from *L* = 1 μM to *L* = 2 μM (A), 10 μM (B), 50 μM (C), and 100 μM (D), with the linearization about the ligand value *L*_0_ = 1 μM. Model parameters are the same as in Figure 9.

We implemented the model as an S-Function in Matlab, version 7 (Mathworks,  http://www.mathworks.com), and linearized it using the *linmod* command. The linearization is valid for even moderately large step changes in ligand concentration (Figure 10). We compared the frequency response of the linearization (Figure 9E) as well as the Barkai-Leibler model [17] (Figure 9F) to experimentally observed frequency responses [20,27]. All frequency responses exhibited low-pass characteristics: low-frequency input components are “passed” through the system and high-frequency input components are attenuated.

For a meaningful quantitative comparison with experimental data, we need to adjust for the fact that the models include only the receptor methylation/demethylation dynamics, but the experimental data measures the system response in terms of motor biases and so contain the dynamics of the phosphorylated CheA, CheY, CheZ, and FliM-bound CheY. To determine the contribution of these elements to the overall frequency response, we used a linearization of the differential equation model for these elements [28]. Finally, because we are primarily interested in the low-pass filtering characteristics, we also accounted for the differentiation. The adjustment was made by combining a system with the same frequency response as in [20] in series with an integrator (to remove the differentiator) and the inverse of the transfer function obtained from a linearization of the phosphorylation cascade modeled in [28] (to remove the phosphorylation cascade that directly affects motor rotation).

The frequency response of the system exhibits characteristics of a differentiator coupled with a first-order low-pass filter; thus, the model of the E. coli signaling network may be approximated by a system with Laplace transform of the form *sk*/(*s* + *k*) where *k* is the bandwidth of the system. The ability of this simple linear system to recreate the response of the model is further verified in Figure 2.

### Environmental model.

**Figure 11:**
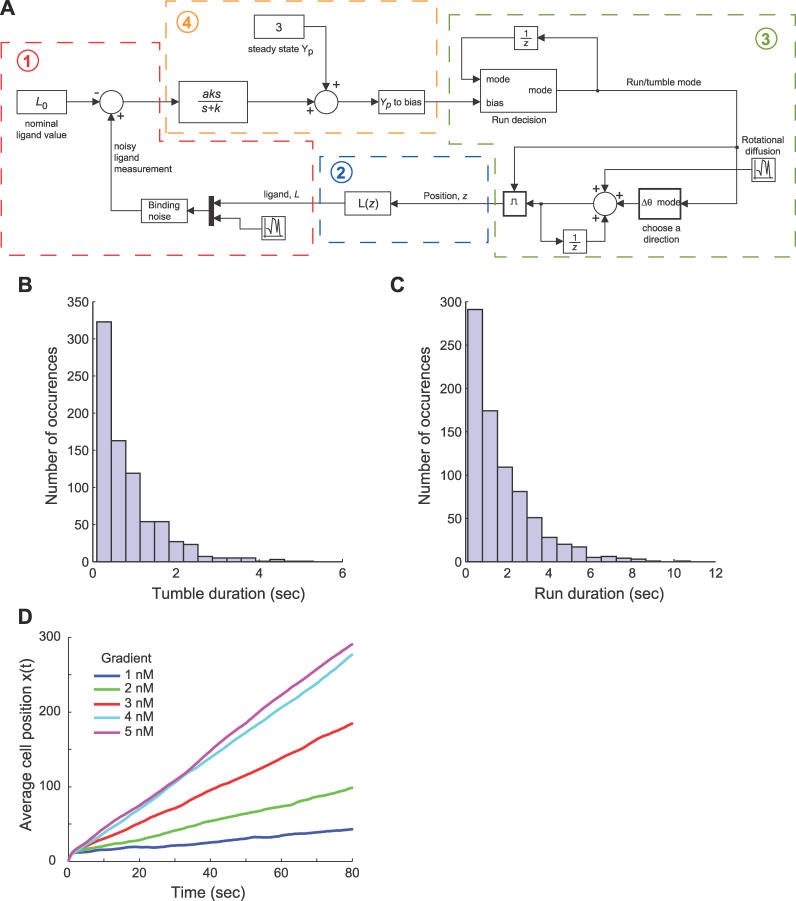
Model of Bacterial Chemotaxis (A) The chemotaxis simulation assumes the following feedback interaction between the bacteria and its environment: 1) the external environment affects the sensing model through the external ligand concentration *L* and incorporates the effect of binding noise; 2) ligand concentration is determined by the location of the cell in the environment (the spatial location *z*); 3) spatial location is determined by integrating the velocity and angle, which incorporates the effect of rotational diffusion and the “run/tumble” decisions; and 4) the run/tumble decisions are based on the signaling mechanism's response (that is, the filter + differentiation) to the measured ligand concentration. (B,C) Validation of chemotaxis model histograms of tumble (B) and run (C) durations for an unstimulated (*g* = *L*_0_ = 0) E. coli with a simulation time of 2,000 s. Both distributions exhibit characteristics of a Poisson process. (D) Average simulation results of 500 runs of a bacteria swimming in a gradient of ligand concentration with varying chemoattractant slopes.

To study the effect of adaptation time on chemotaxis, we developed a computational model of a chemotaxing cell in Simulink version 6.2 by Mathworks, available by contacting the authors; see Figure 11A. We describe the major components of the model here.

### Environment.

Bacteria are assumed to chemotax in a 2-D (*x*,*y*) plane. The ligand concentration at a position *z* = (*x*,*y*) is *L*(*z*) = max(*gx* + *L*_0_,0) where the gradient changes along the *x* direction with slope *g,* and *L*_0_ is a constant initial ligand concentration at *x* = 0. At each simulation step, the concentration of ligand at the bacterium's current location is calculated as *L*(*z*). In the absence of rotational and translational diffusion, cells are assumed to move at a constant velocity of 20 μm/sec. Fluctuations in receptor occupancy are modeled in the simulation by an additive noise term to the ligand concentration. This contribution is assumed normal with distribution *N*(0,*R_T_L*(*z*)*K_D_*/(*K_D_* + *L*(z))^2^) where *K_D_* = *k*_−_/*k*_+_ is the binding dissociation constant, *k*_+_ and *k*_−_ are the on and off rates, respectively, and *R_T_* is the total number of receptors [4,33]. As demonstrated by Berg, even when running the cell cannot maintain a straight path for long because of the effects of rotational diffusion [41]. Rotational diffusion is implemented through an additive stochastic component to the bacterium's heading at each simulation step. This component is assumed normal with distribution *N*(0,2*D_r_t_s_*) where *D_r_* is the rotational diffusion coefficient and *t_s_* is the sample time of the simulation [41]. Translational diffusion is similarly implemented in both the *x* and *y* velocity components with a distribution *N*(0,2*D_t_*/*t_s_*) where *D_t_* is the translational diffusion coefficient. In the latter, *t_s_* appears in the denominator because translational diffusion affects position with a variance of 2*D_t_t_s_* and we are adding a velocity component. The diffusion coefficients used in simulation are *D_r_* = 0.16 radians^2^/second and *D_t_* = 2.2 × 10^−1^ μm^2^/second [41].

### Signal transduction.

The signal transduction pathway in E. coli takes the form of a differentiator coupled with a low-pass filter (see above). We therefore implement the signaling network in simulation with the transfer function *aks*/(*s* + *k*) with gain *a* and cutoff frequency *k*. We assume the system takes ligand as its input and outputs the phosphorylated form of CheY (CheYp) and that this is a linearization of the actual network about a ligand input concentration *L*_0_ and a steady state CheYp concentration of 3 μM [42]. Linearizations of the model suggest a gain of *a* ≈ −0.05, which we used in our simulations. While other gains were found to potentially affect the cutoff frequency optimal for chemotaxis, the existence of an optimal cutoff frequency and the qualitative characteristics of our results, such as the dependence of the optimal cutoff frequency on external parameters, was independent of the gain.

### Running bias.

It is known that the probability of an individual flagellum rotating clockwise, which induces tumbling, or CCW, which leads to runs, is a Hill function of CheYp concentration [42]. We calculate the probability *b* of an individual flagella's rotation being CCW as a function of *Y_p_* using a Hill function with a Hill coefficient of 10 [42]. The parameter *K_m_* is determined using steady-state values of CheYp and a CCW flagellar rotation bias of 3 μM and 0.64, respectively [42]. We assume the majority of a cell's flagella must be rotating CCW for running to occur. Thus, we calculate the probability of running of the entire bacteria as 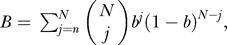 where *N* is the total number of flagella and *n* is the minimum number of CCW-rotating individual flagella needed for running [21]. In our simulations, *N* = 8 and *n* = 5. The decision at each simulation step of whether to run or tumble is made in the “Run decision” simulation block as a function of the current run/tumble state and the flagellar rotation bias that is output from the signaling network. This block essentially determines the probability of running given the current state of the system so that the overall probability of running is given by the bias and the expected run and tumble lengths match those seen in the literature. To achieve this, we use the following analysis. Let *p* be the run bias, *p*_1_ be the run bias given that the cell is already running, and *q*_2_ be the tumble bias given that the cell is already tumbling. The expected run length in number of simulation steps is:

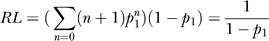
Similarly, the expected tumble length is:

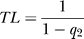
Therefore, the expected run bias is:

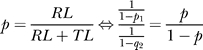



Suppose that *p* and *q*_2_ are fixed. Then, solving for *p*_1_ yields: *p*_1_ = 1 – (1 – *q*_2_) (1 – *p*)/*p*. The expected tumble length is *TL_s_*/*T_s_* steps where *TL_s_* is the expected tumble length in seconds. This can then be converted to a value of *q*_2_: *q*_2_ = 1 – *T_s_*/*TL_s_* which leads to

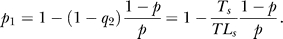



The probabilities *p*_1_ and *q*_2_ are then used to determine at a given simulation step whether the next time step should consist of a run or a tumble.

The tumbling angle θ was assumed to follow the probability density function 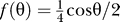 , −π ≤ θ ≤ π [43]. This distribution gives mean tumbling angles of 65° with standard deviation 43° that match the experimentally determined angles closely (68° and 38°, respectively [9]).

The combined signal transduction and environmental model exhibits several traits observed in cells. The exponential shape of the run/tumble distributions is characteristic of a Poisson distribution and is similar to interval distributions found experimentally [44] and in previous models [37] (Figure 11B and 11C). Recent results suggest that, although average run and tumble distributions of a population may exhibit Poisson characteristics, distributions for individual cells may adhere to a power law [5]; however, we assume Poisson characteristics to be a reasonable description of cellular behavior for our purposes. The ability to navigate up a gradient of chemoattractant is evident in that cells travel farther along the *x-*axis (the direction of the gradient) as time progresses (Figure 11D). Furthermore, a larger slope in the gradient of ligand concentration increases the success of the bacteria in swimming in the *x* direction.

### Optimal filter derivation.

To determine the optimal filter for extracting the signal from the noisy measurement, we used Kalman filter theory. The Kalman filter is an algorithm, used widely in engineering navigation and guidance systems, developed to detect and separate signals in the presence of random, unwanted noise [25,26]. General Kalman filtering theory is provided in Protocol S1, and here we present a model of bacterial chemotaxis for the purpose of obtaining accurate chemoattractant estimates using the Kalman filter. Let the state of the system during chemotaxis be

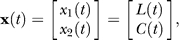
where *L*(*t*) is ligand concentration and *C*(*t*) is the concentration of bound receptor complexes resulting from ligand–receptor binding. The differential equation describing the binding reaction (without noise) is

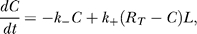
which can be linearized about an average ligand concentration *L*_0_ and a steady-state receptor-complex concentration *C*_0_ to give

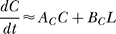
with *A_C_* and *B_C_* constant. Because

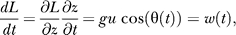
and, if we assume the cell measures *y*(*t*) = *C*(*t*) + *v*(*t*) where the binding noise *v*(*t*) is a zero-mean, white random process with variance *R_T_L*_0_ *K_D_*/(*K_D_* + *L*_0_)^2^ [4,33], then we have the linear system

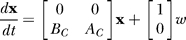


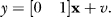



For simplicity, we assume θ(*t*) is a white-noise process with variance 2*D_r_*τ where τ is an average run length and *D_r_* is the rotational diffusion coefficient. This means that *w* is a (nonzero mean) white noise process with variance *g* ^2^ *u* ^2^var[cos(θ(*t*))]. The fact that *w* has nonzero mean can be accounted for by including an input *u*(*t*) and redefining *w*; however, this does not affect the cutoff frequency of the optimal filter.

Our results are obtained by computing the optimal filter for the above system using the *kalman* function from the Control System Toolbox version 6.2 in Matlab. The variance of cos(θ) is approximated numerically in Matlab using an average run length of τ = 1 s.

## Supporting Information

Figure S1 Effect of Filter Rolloff Simulations of chemotactic performance for varying cutoff frequencies were repeated for filters with roll-off of first, second, and third order. Higher-order filters do not affect the existence of an optimal filter cutoff frequency, although the cutoff frequency does decrease. Also, performance is greatest with the first-order filter, possibly due to increased phase delay with the higher-order filters.
(249 KB PDF)

Protocol S1 Kalman Filter Derivation of Optimal Filter (48 KB PDF)

## Abbreviations

CCW: counterclockwise
PSD: power spectral density

## References

[pcbi-0020154-b001] Csete ME, Doyle JC (2002). Reverse engineering of biological complexity. Science.

[pcbi-0020154-b002] Kitano H (2002). Computational systems biology. Nature.

[pcbi-0020154-b003] Rao CV, Wolf DM, Arkin AP (2002). Control, exploitation and tolerance of intracellular noise. Nature.

[pcbi-0020154-b004] Orrell D, Bolouri H (2004). Control of internal and external noise in genetic regulatory networks. J Theor Biol.

[pcbi-0020154-b005] Korobkova E, Emonet T, Vilar JMG, Shimizu TS, Cluzel P (2004). From molecular noise to behavioural variability in a single bacterium. Nature.

[pcbi-0020154-b006] Pedraza JM, van Oudenaarden A (2005). Noise propagation in gene networks. Science.

[pcbi-0020154-b007] Kaern M, Elston TC, Blake WJ, Collins JJ (2005). Stochasticity in gene expression: From theories to phenotypes. Nat Rev Genet.

[pcbi-0020154-b008] Devreotes PN, Zigmond SH (1988). Chemotaxis in eukaryotic cells: A focus on leukocytes and *Dictyostelium*. Annu Rev Cell Biol.

[pcbi-0020154-b009] Berg HC, Brown DA (1972). Chemotaxis in Escherichia coli analysed by three-dimensional tracking. Nature.

[pcbi-0020154-b010] Falke JJ, Bass RB, Butler SL, Chervitz SA, Danielson MA (1997). The two-component signaling pathway of bacterial chemotaxis: A molecular view of signal transduction by receptors, kinases, and adaptation enzymes. Ann Rev Cell Dev Bio.

[pcbi-0020154-b011] Bourret RB, Stock AM (2002). Molecular information processing lessons from bacterial chemotaxis. J Biol Chem.

[pcbi-0020154-b012] Yi TM, Huang Y, Simon MI, Doyle JC (2000). Robust perfect adaptation in bacterial chemotaxis through integral feedback control. Proc Natl Acad Sci U S A.

[pcbi-0020154-b013] Alon U, Surette MG, Barkai N, Leibler S (1999). Robustness in bacterial chemotaxis. Nature.

[pcbi-0020154-b014] Rao CV, Kirby JR, Arkin AP (2004). Design and diversity in bacterial chemotaxis: A comparative study in Escherichia coli and Bacillus subtilis. PLoS Biol.

[pcbi-0020154-b015] Berg HC, Purcell EM (1977). Physics of chemoreception. Biophys J.

[pcbi-0020154-b016] Strong SP, Freedman B, Bialek W, Koberle R (1998). Adaptation and optimal chemotactic strategy in E. coli. Phys Rev E.

[pcbi-0020154-b017] Barkai N, Leibler S (1997). Robustness in simple biochemical networks. Nature.

[pcbi-0020154-b018] Mello BA, Tu Y (2005). An allosteric model for heterogeneous receptor complexes: Understanding bacterial chemotaxis responses to multiple stimuli. Proc Natl Acad Sci U S A.

[pcbi-0020154-b019] Keymer JE, Endres RG, Skoge M, Meir Y, Wingreen NS (2006). Chemosensing in Escherichia coli: Two regimes of two-state receptors. Proc Natl Acad Sci U S A.

[pcbi-0020154-b020] Block SM, Segall JE, Berg HC (1982). Impulse responses in bacterial chemotaxis. Cell.

[pcbi-0020154-b021] Spiro PA, Parkinson JS, Othmer HG (1997). A model of excitation and adaptation in bacterial chemotaxis. Proc Natl Acad Sci U S A.

[pcbi-0020154-b022] Gillespie DT (2000). The chemical Langevin equation. J Chem Phys.

[pcbi-0020154-b023] DeLisi C, Marchetti F, Del Grosso G (1982). A theory of measurement error and its implications for spatial and temporal gradient sensing during chemotaxis. Cell Biophys.

[pcbi-0020154-b024] Bialek W, Setayeshgar S (2005). Physical limits to biochemical signaling. Proc Natl Acad Sci U S A.

[pcbi-0020154-b025] Kalman RE (1960). A new approach to linear filtering and prediction problems. Trans ASME J Basic Eng.

[pcbi-0020154-b026] Kalman RE, Bucy R (1961). New results in linear filtering and prediction theory. Trans ASME J Basic Eng.

[pcbi-0020154-b027] Segall JE, Block SM, Berg HC (1986). Temporal comparisons in bacterial chemotaxis. Proc Natl Acad Sci U S A.

[pcbi-0020154-b028] Sourjik V, Berg HC (2002). Binding of the Escherichia coli response regulator CheY to its target measured in vivo by fluorescence resonance energy transfer. Proc Natl Acad Sci U S A.

[pcbi-0020154-b029] Ma L, Janetopoulos C, Yang L, Devreotes PN, Iglesias PA (2004). Two local excitation, global inhibition mechanisms acting complementarily in parallel can explain the chemoattractant-induced PI(3,4,5)P_3_ response in *Dictyostelium*. Biophys J.

[pcbi-0020154-b030] Janetopoulos C, Ma L, Devreotes PN, Iglesias PA (2004). Chemoattractant-induced phosphatidylinositol 3,4,5-trisphosphate accumulation is spatially amplified and adapts, independent of the actin cytoskeleton. Proc Natl Acad Sci U S A.

[pcbi-0020154-b031] Fisher PR, Merkl R, Gerisch G (1989). Quantitative analysis of cell motility and chemotaxis in Dictyostelium discoideum by using an image processing system and a novel chemotaxis chamber providing stationary chemical gradients. J Cell Biol.

[pcbi-0020154-b032] Berg HC (1988). A physicist looks at bacterial chemotaxis. Cold Spring Harb Symp Quant Biol.

[pcbi-0020154-b033] Lauffenburger DA, Linderman JL (1993). Receptors.

[pcbi-0020154-b034] Carlson JM, Doyle J (2002). Complexity and robustness. Proc Natl Acad Sci U S A.

[pcbi-0020154-b035] Mello BA, Tu Y (2003). Quantitative modeling of sensitivity in bacterial chemotaxis: The role of coupling among different chemoreceptor species. Proc Natl Acad Sci U S A.

[pcbi-0020154-b036] Shimizu TS, Aksenov SV, Bray D (2003). A spatially extended stochastic model of the bacterial chemotaxis signalling pathway. J Mol Biol.

[pcbi-0020154-b037] Bray D, Bourret RB, Simon MI (1993). Computer simulation of the phosphorylation cascade controlling bacterial chemotaxis. Mol Biol Cell.

[pcbi-0020154-b038] Sourjik V, Berg HC (2002). Receptor sensitivity in bacterial chemotaxis. Proc Natl Acad Sci U S A.

[pcbi-0020154-b039] Sourjik V, Berg HC (2004). Functional interactions between receptors in bacterial chemotaxis. Nature.

[pcbi-0020154-b040] Mello BA, Shaw L, Tu Y (2004). Effects of receptor interaction in bacterial chemotaxis. Biophys J.

[pcbi-0020154-b041] Berg HC (1993). Random walks in biology.

[pcbi-0020154-b042] Cluzel P, Surette M, Leibler S (2000). An ultrasensitive bacterial motor revealed by monitoring signaling proteins in single cells. Science.

[pcbi-0020154-b043] Dillon R, Fauci L, Gaver D III (1995). A microscale model of bacterial swimming, chemotaxis and substrate transport. J Theor Biol.

[pcbi-0020154-b044] Block SM, Segall JE, Berg HC (1983). Adaptation kinetics in bacterial chemotaxis. J Bacteriol.

