# Supplementary material for: Optimal Noise Filtering in the Chemotactic Response of Escherichia coli
Source: PLoS Comput Biol. 2006 Nov 17;2(11):e154. doi: 10.1371/journal.pcbi.0020154 (PMC1636674; doi:10.1371/journal.pcbi.0020154)
Supplement: Supplementary file 1 [file pcbi.0020154.sd001.pdf]

Supplementary Material

The Kalman filter

The Kalman filter is an algorithm, used widely in engineering navigation and guidance systems, developed to detect and separate signals in the presence of random, unwanted noise [1, 2]. Here we present the continuous-time version of the Kalman filter (the Kalman-Bucy filter) as described in [3].

Consider a linear system with state $x(t)$ and measurement $y(t)$:

$$\dot{x}(t) = Fx(t) + Bu(t) + Gw(t) \quad (1)$$
$$y(t) = Hx(t) + Du(t) + v(t) \quad (2)$$

where $x(t)$, $u(t)$, $w(t)$, and $v(t)$ are vectors of known dimensions and the matrices F, B, H, D and G have dimensions corresponding to the vector dimensions. The process noise $w(t)$ and measurement noise $v(t)$ are assumed zero-mean white noise processes with $E[w(t)] = 0$, $E[v(t)] = 0$, $E[w(t)w^\top(\tau)] = Q\delta(t-\tau)$, $E[v(t)v^\top(\tau)] = R\delta(t-\tau)$, and $E[w(t)v^\top(\tau)] = 0$. The covariance matrices Q and R are positive semidefinite and definite, respectively. We would like to obtain an estimate $\hat{x}$ of x given the observation y. This optimal estimator is given by the Kalman Filter:

$$\dot{\hat{x}} = F\hat{x} + Bu + K(y - H\hat{x} - Du),$$

where $K = PH^\top R^{-1}$ is the Kalman gain matrix and the initial state is $\hat{x}_0 = E[x(t_0)]$. Note that $\hat{x}$ follows the same dynamics as x adjusted by the innovation gained from comparing the current estimate of the output with the actual measurement. The covariance matrix P is governed by the matrix Riccati differential equation

$$\dot{P} = FP + PF^T - PH^T R^{-1} HP + GQG^\top,$$

and is initialized with $P(t_0) = E[(x(t_0) - E[x(t_0)])(x(t_0) - E[x(t_0)])^\top]$. Solving $\dot{P} = 0$ yields the steady-state filter.

If we have the simple scalar system $\dot{x} = w$, $y = x + v$, then solving the steady-state Riccati equation yields $P^2 = QR$ and $K = \sqrt{Q/R}$. The Kalman estimator is then

$$\dot{\hat{x}} = \sqrt{\frac{Q}{R}}(y - \hat{x}).$$

This is a low pass filter with bandwidth equal to the signal-to-noise ratio Q/R as seen by obtaining the transfer function from y to $\hat{x}$:

$$\frac{\hat{X}(s)}{Y(s)} = \frac{\sqrt{Q/R}}{s + \sqrt{Q/R}}.$$

References

[1] Kalman RE (1960) A new approach to linear filtering and prediction problems. Trans ASME J Basic Eng Series D 82:35–46.

[2] Kalman RE, Bucy R (1961) New results in linear filtering and prediction theory. Trans ASME J Basic Eng Series D 83:95–108.

[3] Mendel JM (1995) Lessons in Estimation Theory for Signal Processing, Communications, and Control. Upper Saddle River, N.J.: Prentice Hall.
