# Supplementary material for: Optimal Noise Filtering in the Chemotactic Response of Escherichia coli
Source: PLoS Comput Biol. 2006 Nov 17;2(11):e154. doi: 10.1371/journal.pcbi.0020154 (PMC1636674; doi:10.1371/journal.pcbi.0020154)
Supplement: Supplementary file 2 [file pcbi.0020154.sg001.pdf]

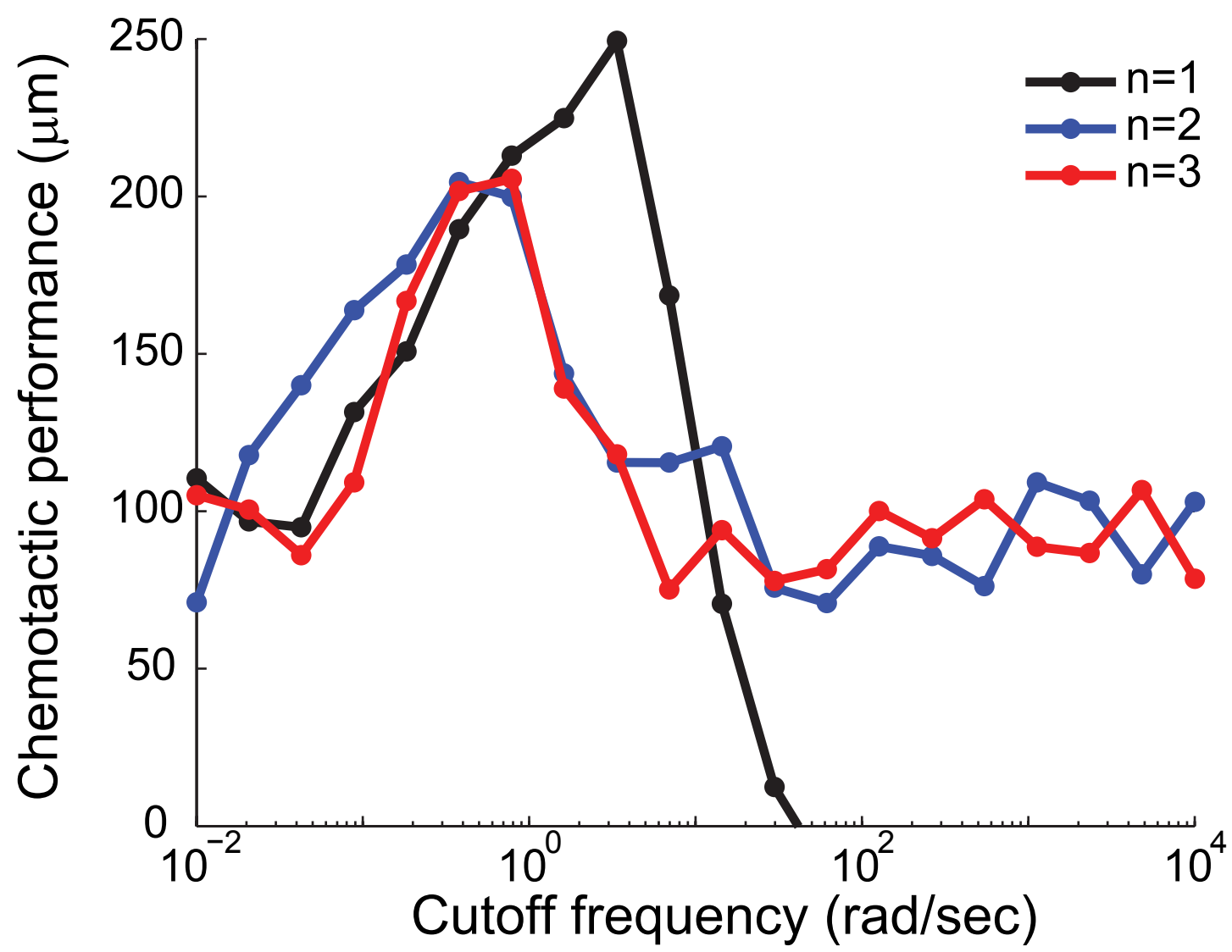

Chemotactic performance (μm)
250
200
150
100
50
0
10^−2
10^0
10^2
10^4
Cutoff frequency (rad/sec)
n=1
n=2
n=3
